# Changes in the Distribution of *Botrytis cinerea* Pers. Fr. In China Under Climate Change

**DOI:** 10.1002/ece3.71640

**Published:** 2025-07-09

**Authors:** Qianqian Qian, Zhihang Zhuo, Yaqin Peng, Danping Xu

**Affiliations:** ^1^ College of Life Science China West Normal University Nanchong China

**Keywords:** *Botrytis cinerea*, climate change, MaxEnt model, potential geographical distribution

## Abstract

*Botrytis cinerea* Pers. Fr. is capable of infecting many horticultural plants and agricultural products with gray mold, which causes great losses to the world economy. MaxEnt is a probabilistic model for classification and prediction. In this study, the MaxEnt model was used to predict the current and future potential geographic distribution of *B. cinerea* in China, and key environmental variables affecting its distribution were identified. The results showed that under the current climatic conditions, the central area of suitable distribution of *B. cinerea* is in Gande County, Guoluo Tibetan Autonomous Prefecture, Qinghai Province, China (99.63° E, 33.92° N). The highly suitable areas are mainly concentrated in tropical and subtropical regions, including Tianjin, Shandong, Anhui, Hubei, and Henan in China. Under the future climate conditions, the center of the suitable distribution of *B. cinerea* did not shift significantly. The areas of both the high and low suitable areas of *B. cinerea* decreased, but the areas of the medium suitable areas increased. Key environmental variables affecting the distribution of *B. cinerea* included isothermality (bio3), mean temperature of wettest quarter (bio8), mean temperature of driest quarter (bio9), precipitation seasonality (bio15), precipitation of coldest quarter (bio19), and elevation. This study has the potential to be utilized to understand the changing patterns of *B. cinerea* distribution and to promote ecological conservation and agricultural management.

## Graphical abstract

The current center of mass of *Botrytis cinerea* is at (99.63° E, 33.92° N). Temperature and precipitation as key environmental variables influencing the distribution of Botrytis cinerea.

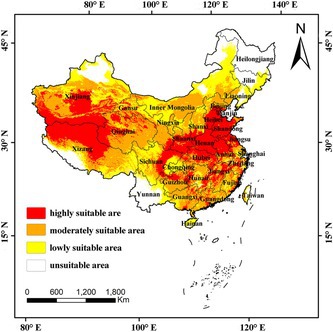

## 1 Introduction

*Botrytis cinerea* Pers. Fr. causes gray mold and belongs to the family Sclerotiniaceae (Da Silva Ripardo‐Filho et al. 2023). There are more than 35 species in the genus *Botrytis*, of which *B. cinerea* is the best known and is considered the second most important plant pathogen in molecular plant pathology (Bi et al. 2023; Garfinkel 2020; Orozco‐Mosqueda et al. 2023). It is capable of infecting more than 600 vascular plant genera and about 1400 plant species and is one of the most widely studied plant pathogens (Boddy 2016; Da Silva Ripardo‐Filho et al. 2023; Orozco‐Mosqueda et al. 2023; Williamson et al. 2007). *B. cinerea* is a necrotrophic pathogen, so damaged and senescent tissues are more susceptible to infection (Hua et al. 2018; Petrasch et al. 2019). Plants can be infected by *B. cinerea* both during the pre‐harvest (growth stage) and post‐harvest phases, with stems, leaves, flowers, fruits, and seeds all serving as potential targets for pathogen colonization (Bi et al. 2023). At the same time, *B. cinerea* is able to survive at low temperatures, thus posing a greater threat to vegetables, melons, and fruits in storage (De Simone et al. 2020). *B. cinerea* is capable of infecting horticultural plants such as roses (Quijada‐Morin et al. 2018), with economically important crops including kiwifruit ( *Actinidia Chinensis* ) (Li et al. 2023), strawberries (Jia et al. 2020), and grapes (Harper et al. 2021) being particularly susceptible, leading to substantial losses in global agricultural production (Perez‐Lara et al. 2023). In Australia, *B. cinerea* affects almost all vineyard areas, causing on average up to A$50 million in losses to the grape and wine industry each year (Harper et al. 2021). Globally, this fungal pathogen is responsible for economic losses exceeding US$10 billion (Cheung et al. 2020). *B. cinerea* is mainly found in temperate and subtropical regions (Williamson et al. 2007). Apple, a fruit widely grown in temperate regions, is infested with *B. cinerea* after harvest, which severely affects apple quality and yield (Leng et al. 2022). The risk of *B. cinerea* infection is also greatly increased by the use of greenhouses (Williamson et al. 2007). Future climate change creates uncertainty for the spread of fungal diseases (Rienth et al. 2021). For example, increased precipitation alters air temperature and relative humidity, which increases the incidence of fungal diseases (Montes et al. 2022). So this study would predict the potential geographic distribution of *B. cinerea* through the maximum entropy (MaxEnt) model.

The maximum entropy (MaxEnt) model is an ecological niche modeling approach that utilizes species occurrence records and environmental variables to predict potential species distribution patterns through machine learning algorithms (Venne and Currie 2021). There are many common species distribution models, including Bioclim, GARP, Climate, and others (Li et al. 2022). These models, based on real data and mapping tools, objectively analyze the spatial patterns of species presence and assess the presence of potentially suitable areas based on environmental characteristics (Mateo et al. 2011). The MaxEnt model has become one of the most widely used approaches for predicting species potential distributions due to its superior predictive accuracy and user‐friendly operation (Gao et al. 2023). Furthermore, it makes good predictions of species distributions when only species occurrence records are available and sample sizes are small (Deng et al. 2022). The MaxEnt model is widely used to predict the distribution of plants and insects around the globe. Zhuo et al. (2020) used the MaxEnt model to simulate the current and future distribution of *Zanthoxylum bungeanum* Maxim. in China. Mahatara et al. (2021) simulated the suitable habitat of the endangered tree species *Dalbergia latifolia* Roxb in Nepal. Dahal et al. (2023) assessed the suitable habitat of *Aceros nipalensis* 's current and future suitable habitat in Bhutan. Similarly, the MaxEnt model has been applied to predict the distribution of microorganisms (Alkhalifah et al. 2022). It predicted globally suitable habitats for black mold *Aspergillus niger* under several climatic scenarios (Wei et al. 2021) and assessed the current potential geographic distribution of caterpillar fungus and future distribution under different climatic scenarios.

The Sixth Assessment Synthesis Report, the Intergovernmental Panel on Climate Change (IPCC, https://www.ipcc.ch/assessment‐report/ar6/) confirmed that continued greenhouse gas emissions will lead to a further increase in global temperatures. Many studies have shown that climate change affects suitable habitats for fungi (Guo et al. 2017; Li et al. 2022). In this study, we simulated the current potential distribution of *B. cinerea* in China by MaxEnt model and predicted the suitable distribution areas under the Shared Socioeconomic Pathways (SSPs) for the future periods of the 2050s and 2090s. This study provides a basis for predicting the distribution of *B. cinerea* under different climate change conditions and a theoretical basis for preventing diseases caused by *B. cinerea* .

## 2 Materials and Methods

### 2.1 Species Occurrence Data

The construction of species distribution models requires adequate occurrence records (Li et al. 2022). The occurrence records of *B. cinerea* used in this study were obtained from the Global Biodiversity Information Facility (GBIF, http://www.gbif.org/). According to GBIF records, *B. cinerea* has been documented in multiple countries, including Japan, Zambia, Israel, Korea, and China. In this study, we systematically compiled the occurrence records with precise geographic coordinates (latitude and longitude) from these data. The data were then preprocessed, that is, duplicates were removed, and it was ensured that only one occurrence record appeared per 1 km grid cell (Zhao et al. 2022). This was done by calculating the distance between the distribution points and the center of the censored grid through the spatial analysis function of ArcGIS and removing overfitted data (Wang et al. 2021). In this study, 138 occurrence records were finally retained and saved in .csv format for modeling.

### 2.2 Environmental Variables

**FIGURE 1:**
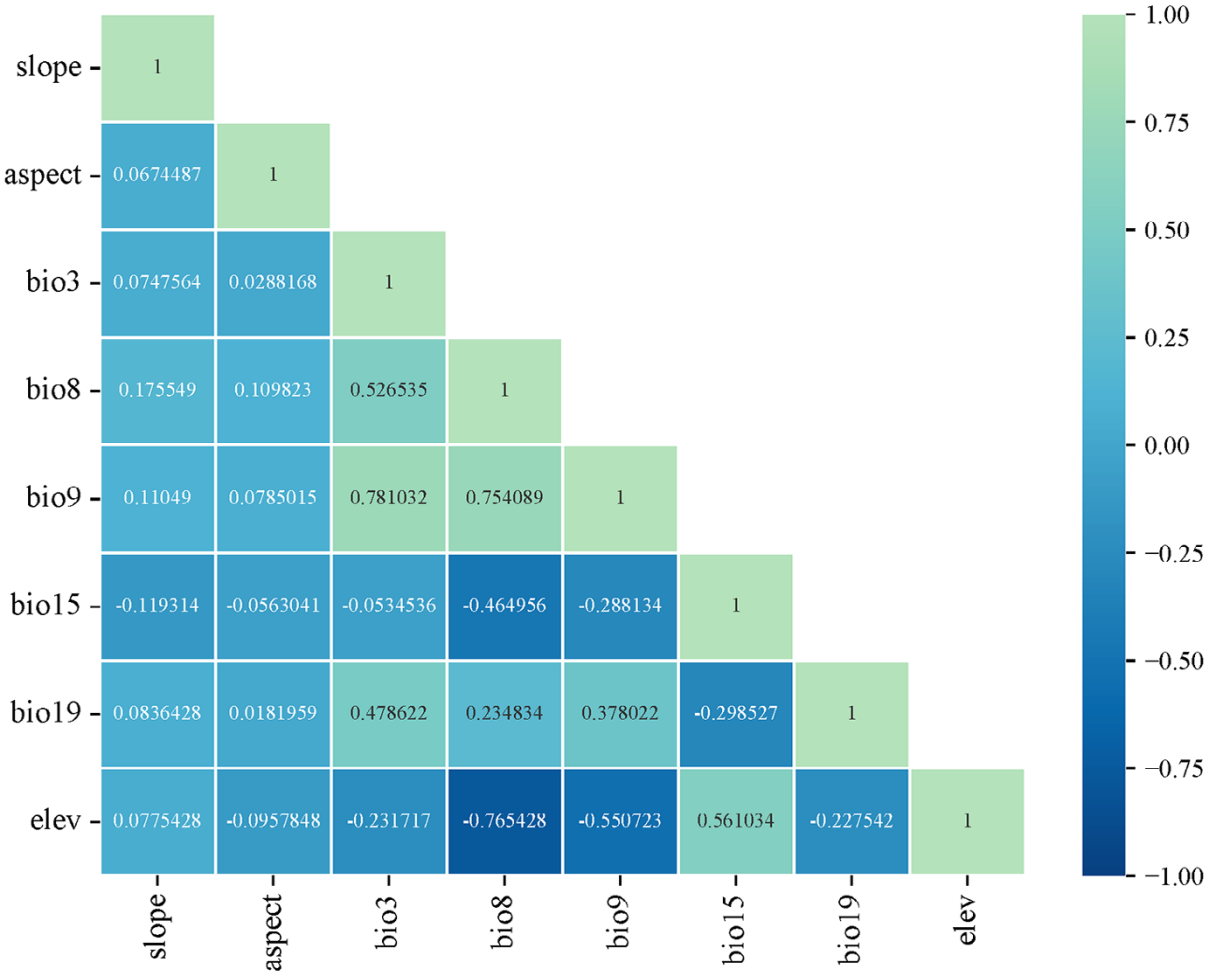
Environment variables for |*r*| ≤ 0.8.

In this study, 19 bioclimatic variables (2.5 arc‐min resolution, ~5 km) were downloaded from WorldClim 2.1 (https://worldclim.org/) (Li et al. 2022), and three topographic datasets (Table S1) were downloaded from the National Oceanic and Atmospheric Administration's National Centers for Environmental Information (NOAANCEI, https://www.ngdc.noaa.gov/) for initial model construction (Table S1). The Shared Socio‐Economic Pathways (SSPs) are a new generation of scenario portfolios constructed by the IPCC to facilitate integrated analysis of future climate change adaptation and mitigation. In this study, three scenarios, SSP1‐2.6 (sustainable development path), SSP3‐7.0 (partial development path) and SSP5‐8.5 (conventional development path) were used to predict suitable distribution areas for *B. cinerea* in the 2050s and 2070s (Li et al. 2022). The 138 occurrence records and all environmental variables data were first imported into MaxEnt 3.4.4 software, and then 25% of the occurrence records were randomly selected as the test set, and the remaining 75% of the occurrence records were used as the training set, and the run was repeated 10 times (Li et al. 2022). In order to avoid having multicollinearity between environmental variables, which would lead to model overfitting, the Variance Inflation Factor (VIF) in IBM SPSS Statistics 27 was used to test for covariance, and environmental factors with VIF > 100 were excluded (Liao et al. 2024). After that, Pearson correlation between environmental variables was calculated by the “*corrplot*” package of R v4.0.4 software (Li et al. 2022), and variables with correlation |*r*| > 0.8 were removed (Figure 1). Based on the results of the run, environmental variables with a contribution rate < 1 were removed (Table 1), and the remaining were identified as key environmental variables affecting the distribution of *B. cinerea* .

**TABLE 1:** Environment variables and their contributions.

Environmental variable	Description	Percent contribution	Permutation importance
Bio3	Isothermality (Bio2/Bio7) (×100)	38.1	18.6
Bio8	Mean temperature of the wettest quarter	24.8	44.4
Elev	Elevation	19.4	4.6
Bio9	Mean temperature of the driest quarter	12	20.9
Bio19	Precipitation of the coldest quarter	2.8	3.5
Bio15	Precipitation seasonality (coefficient of variation)	1.6	7.6
Slope	—	0.9	0.3
Aspect	—	0.3	0

### 2.3 Maxent Model Building and Evaluation

In order to avoid the incompatibility of MaxEnt default parameters for different species, the model was optimized in this paper using the R package *ENMeval* 2.0.5. Re‐modeling based on the filtered key environmental variables, 25% of the occurrence records are still selected as the test set, and the remaining 75% of the occurrence records are the training set, and the run is repeated 10 times. The results were validated using 10‐fold cross‐validation. The model performance is evaluated by the ROC curve, and the sum of the areas under the curve (AUC value) is used to indicate the accuracy of the model. The AUC value between 0 and 0.6 indicates that the model is failing; 0.6–0.7 indicates that the model is poor; 0.7–0.8 indicates that the model is fair; 0.8–0.9 indicates that the model is good; and 0.9–1 indicates that the model is excellent. The Jackknife test was used to measure the importance of key environmental variables. The response of *B. cinerea* to key environmental variables was represented by response curves. The distribution probability of species was visualized through ArcGIS. In order to clearly express the results, the probability of species presence (P) was divided into four classes, which were distinguished by different colors on the map: highly suitable area (*p* ≥ 0.6, red), moderately suitable area (0.4 ≤ *p* < 0.6, orange), lowly suitable area (0.4 ≤ *p* < 0.2, yellow) and unsuitable area (*p* < 0.2, white).

## 3 Results

### 3.1 Model Results Validation

**FIGURE 2:**
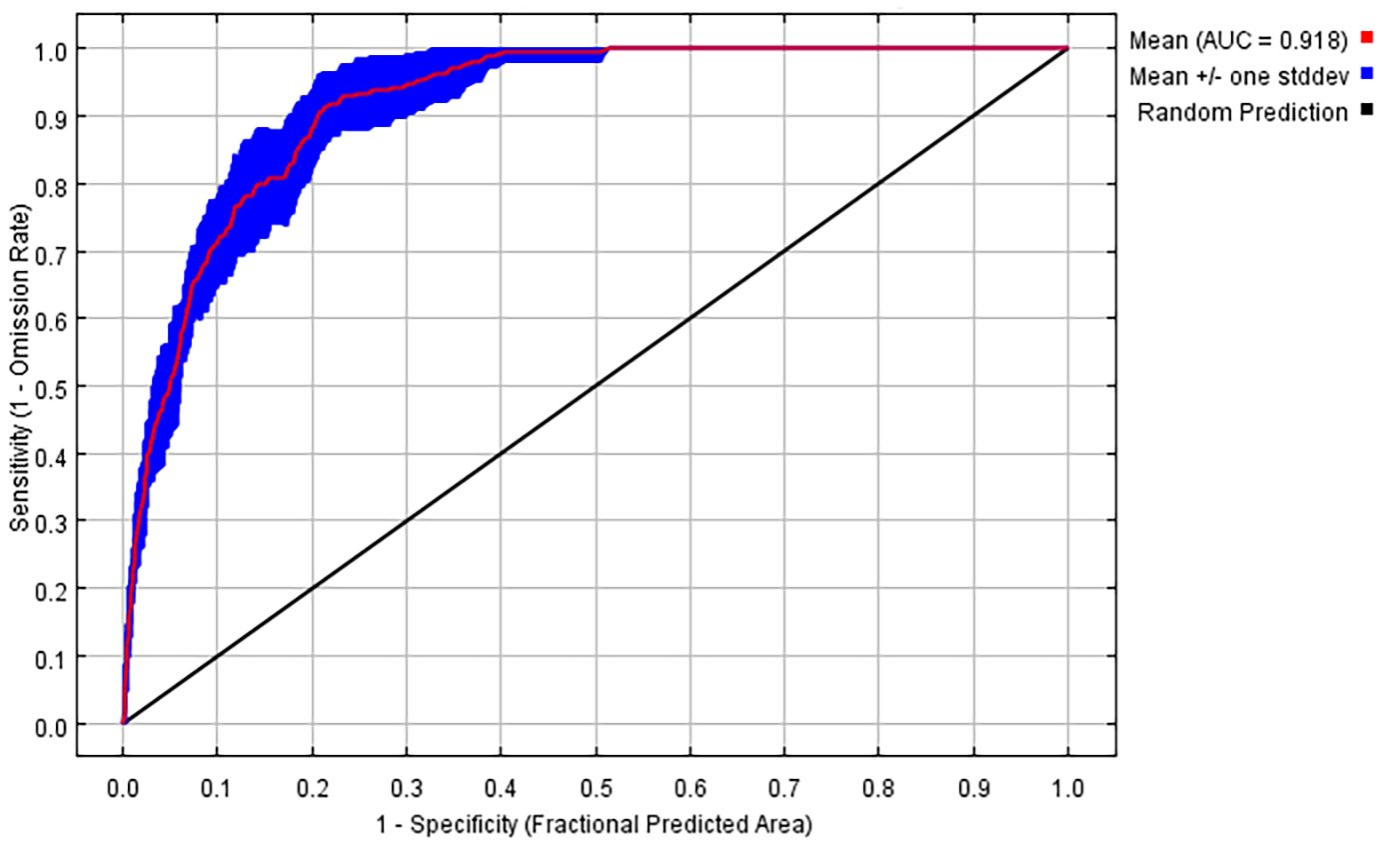
The area under the ROC curve (AUC) for *Botrytis cinerea*.

By evaluating different combinations of feature combination (FC) and regularization multiplier (RM) parameters, the combination of FC = 0.8, RM = LPH was chosen for modeling. The sum of areas under the curve (AUC value) of ROC was used to test the accuracy of the model. The AUC value obtained after running the model is 0.918 (Figure 2), which is greater than 0.9, indicating that the model predicts the results with high accuracy.

### 3.2 The Current Distribution of *Botrytis cinerea*

**FIGURE 3:**
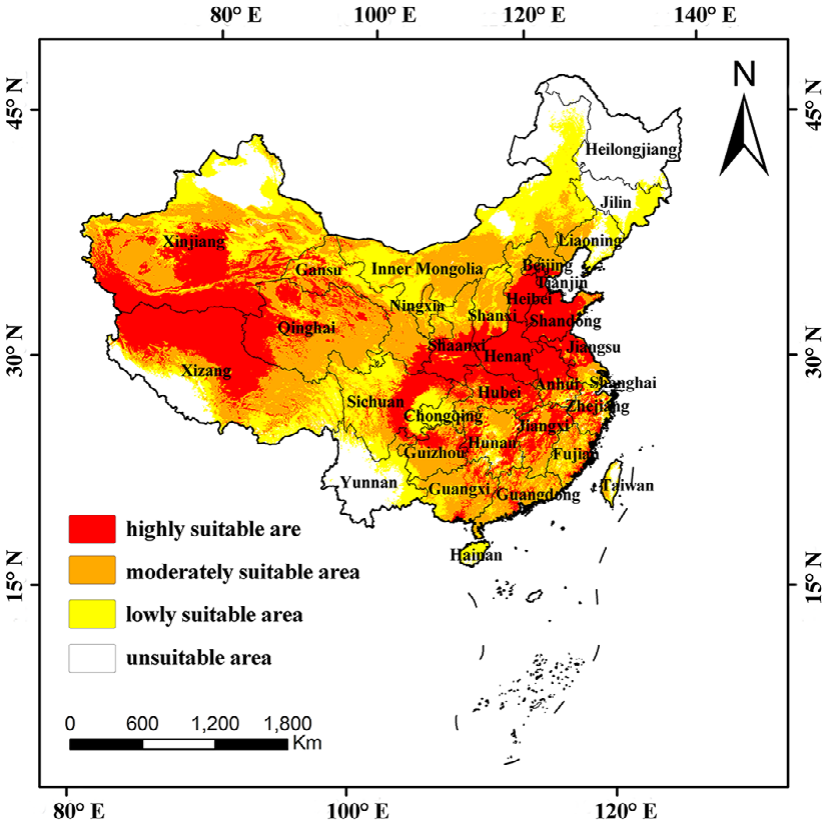
Current potential distribution of *Botrytis cinerea*.

Four different colors in the map were used to distinguish the high, medium, low, and unsuitable areas of *B. cinerea* (Figure 3). The results show that *B. cinerea* can survive in most of the provinces in China under the current climatic conditions, and the high suitability zones are mainly concentrated in the mid‐latitude areas such as Central China, East China, Huaihe River Basin, Haihe River Basin, and Xizang Endorheic Region. Analyzing each province individually (Table 2), it was found that the highly suitable areas were mainly located in Tianjin, Shandong, Anhui, Hubei, and Henan. The proportion of highly suitable areas in these three provinces exceeded 60% of the province's area, with Henan having the highest proportion of 96.11%. Beijing, Jiangsu, Zhejiang, Jiangxi, Xizang, Qinghai and Xinjiang also have more highly suitable areas, accounting for more than 30% of the province's area. Inner Mongolia, Liaoning, Yunnan, Ningxia, and Taiwan have less distribution of highly suitable areas, with the percentage of all of them being less than 5% of the province's area. The area of highly suitable areas in China reaches 261.15 × 10^4^ km^2^, accounting for 27.20% of the land area of China. The area of medium‐suitable zones in China as a whole is 307.81 × 10^4^ km^2^, mainly distributed in central Inner Mongolia, Guangdong, Guangxi, northwestern Xizang, western Qinghai, and southern Xinjiang. There are 228.28 × 10^4^ km^2^ of low‐suitable areas in China, mainly distributed in eastern Xizang, western Sichuan, and southern Chongqing. The area of unsuitable zones reaches 163.88 × 10^4^ km^2^, with the unsuitable zones in Heilongjiang and Yunnan covering almost the whole province, followed by a large number of unsuitable zones in Liaoning, Jilin, northern Xinjiang, and eastern Inner Mongolia. The provinces of Beijing, Tianjin, Hebei, Shanxi, Shanghai, Jiangsu, Shandong, Qinghai, Ningxia, and Hong Kong do not have any unsuitable areas, and Tianjin, Shandong, and Hong Kong all have high and medium suitable areas.

**TABLE 2:** Analysis of main suitable distributions of *Botrytis cinerea*.

Province	High suitable area (10^4^ km^2^)	Total (10^4^ km^2^)	Percentage of high suitable area in province (%)	Percentage of high suitable area in China (%)
Beijing	0.72	1.64	43.83	0.07
Tianjin	1.10	1.19	92.06	0.11
Hebei	8.79	64.44	13.65	0.92
Shanxi	2.86	15.67	18.24	0.30
Inner Mongolia	0.31	118.30	0.26	0.03
Liaoning	0.03	14.59	0.21	0.00
Jiangsu	5.97	10.72	55.71	0.62
Zhejiang	3.18	10.18	31.26	0.33
Anhui	9.64	13.9	69.35	1.00
Fujian	1.74	12.14	14.32	0.18
Jiangxi	7.61	16.69	45.59	0.79
Shandong	13.80	15.67	88.09	1.44
Henan	16.05	16.70	96.11	1.67
Hubei	11.63	18.59	62.58	1.21
Hunan	5.76	21.18	27.21	0.60
Guangdong	2.39	17.98	13.31	0.25
Guangxi	2.70	23.76	11.36	0.28
Chongqing	1.47	8.24	17.82	0.15
Sichuan	12.22	48.5	25.20	1.27
Guizhou	3.64	17.62	20.64	0.38
Yunnan	0.82	39.41	2.08	0.09
Xizang	47.94	122.84	39.02	4.99
Shaanxi	11.80	20.58	57.34	1.23
Gansu	8.51	45.40	18.73	0.89
Qinghai	23.60	69.67	33.87	2.46
Ningxia	0.02	6.64	0.26	0.00
Xinjiang	56.83	166.49	34.13	5.92
Taiwan	0.00	3.59	0.10	0.00
Hong Kong	0.02	0.11	18.94	0.00
China (land area)	261.15	960	—	27.20

### 3.3 Distribution and Change of Future Potential Suitable Habitat

**FIGURE 4:**
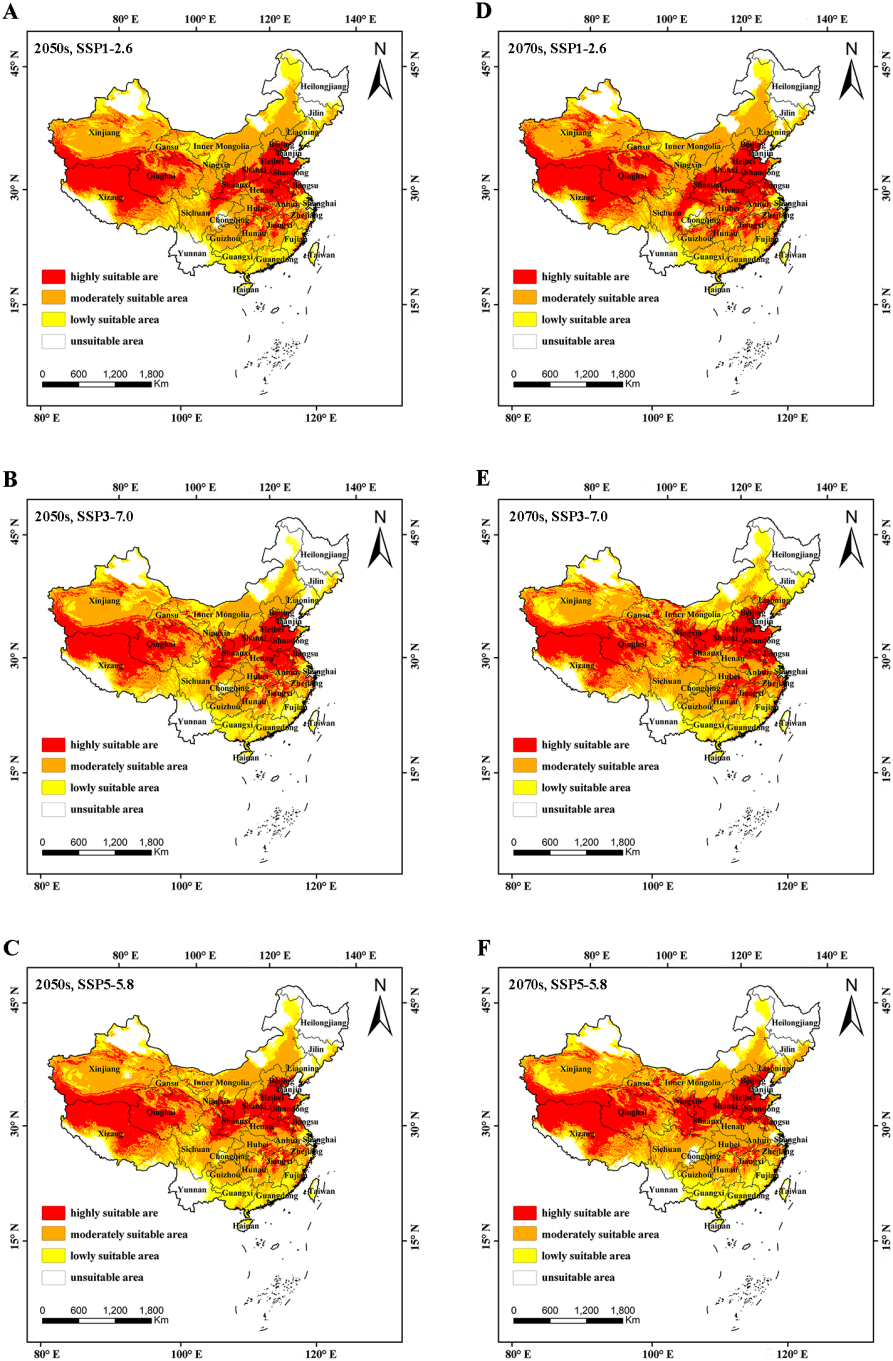
Future distribution of *Botrytis cinerea*. (A) 2050s, SSP1‐2.6; 2050s, (B) SSP3‐7.0; 2050s, (C) SSP5‐5.8; (D) 2070s, SSP1‐2.6; (E) 2070s, SSP3‐7.0; (F) 2070s, SSP5‐5.8.

In order to predict the change of future potential suitable habitat of *B. cinerea* in China, two periods of 2050s and 2070s were chosen to simulate the change of *B. cinerea* under the climatic scenarios of SSP1‐2.6, SSP3‐7.0, and SSP5‐5.8, respectively (Figure 4). In this study, we compared the changes in the area of suitable areas for *B. cinerea i*n China between the current period and different future periods. The results showed (Table 3) that in the 2050s, the area of both high and low suitable areas decreased under the three climate scenarios, with the area of low suitable areas decreasing the most under the SSP5‐8.5 scenario, by 20.11%. In the 2070s, the area of low suitability zones decreased under all three climate scenarios, with the largest decrease of 34.38% under scenario SSP1‐2.6. High suitability zones in the 2070s showed an increase under scenarios SSP1‐5.6 and SSP3‐7.0, with an increase of 13.41% and 0.90%, respectively. It is noteworthy that the area of the medium suitability zone showed an increase in all periods and scenarios, with the highest increase (20.06%) in the 2050s under the SSP1‐2.6 scenario and the lowest increase (1.32%) in the 2070s under the SSP3‐7.0 scenario.

**TABLE 3:** Predicted suitable areas for *Botrytis cinerea* under current and future climatic conditions.

Decade	Scenarios	Predicted area (10^4^ km^2^)			Comparise with current distribution (%)		
		High suitable area	Moderate suitable area	Low suitable area	High suitable area	Moderate suitable area	Low suitable area
Current	—	261.15	307.81	228.28	—	—	—
2050s	SSP1‐2.6	248.05	369.57	187.70	−5.02	20.06	−17.78
	SSP3‐7.0	245.87	325.51	193.85	−5.85	5.75	−15.08
	SSP5‐8.5	249.90	350.88	182.38	−4.31	13.99	−20.11
2070s	SSP1‐2.6	296.16	352.00	149.79	13.41	14.36	−34.38
	SSP3‐7.0	263.51	311.86	213.20	0.90	1.32	−6.61
	SSP5‐8.5	257.12	331.07	176.36	−1.54	7.56	−22.74

### 3.4 Analysis of Environmental Variables

**FIGURE 5:**
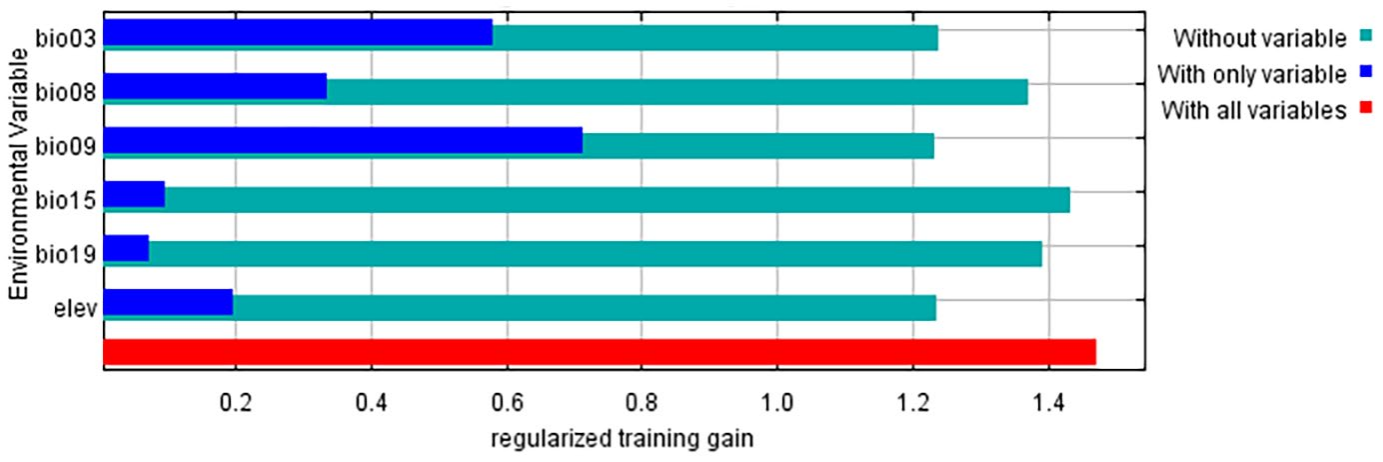
The importance of environment variables to *Botrytis cinerea*.

**FIGURE 6:**
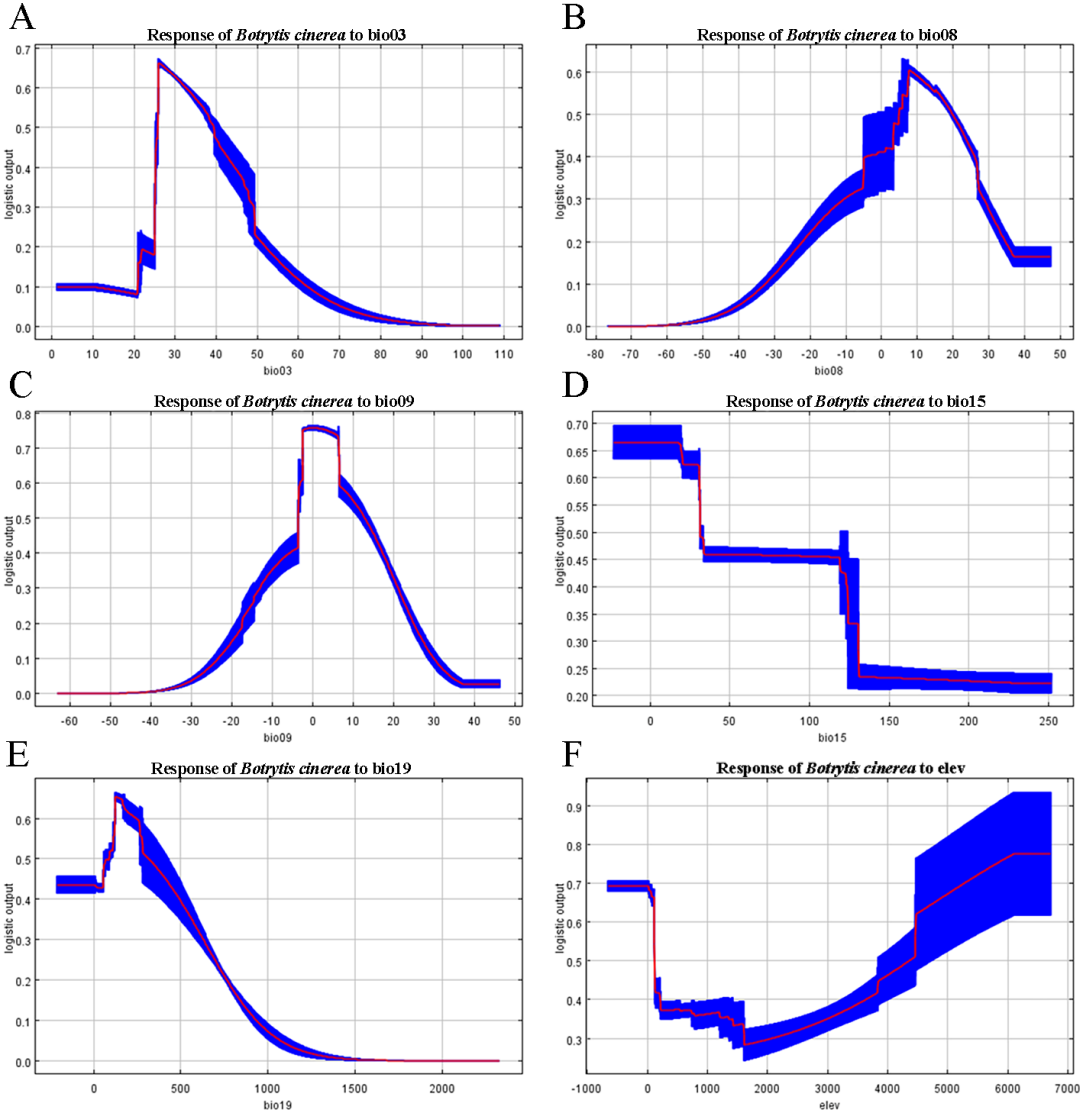
Response curve of environmental variables to occurrence probability of *Botrytis cinerea*. (A) bio 3; (B) bio 8; (C) bio 9; (D) bio 15; (E) bio 19; (F) elev.

We identified the key environmental variables affecting the distribution of *B. cinerea* by contribution ratio and Pearson correlation, and analyzed the regularized training gain of the key environmental variables by jackknife (Figure 5). The blue bars (With only variable) represent the model's predictive performance when using solely that variable (with other variables excluded). The taller the blue bar, the greater the individual contribution of that variable to the model. The green bars (Without variable) indicate the model's performance when excluding that particular variable (while retaining all others). The shorter the green bar, the more critical that variable's contribution is. The red bar (With all variables) shows the model's performance using all variables (serving as the baseline value), which is used to compare the blue and green bars to assess each variable's independent and dependent contributions. Bio9 exhibits the longest blue bar and shortest green bar, demonstrating it is the most crucial environmental variable influencing *B. cinerea* distribution, followed by bio3. The appropriate range of the effect of each key environmental variable on the distribution of *B. cinerea* (probability of existence: *p* ≥ 0.6) was shown by the response curves (Figure 6). The results showed that the optimum range of isothermality (bio3) was 25.94%–33.31%, peaking at 27.28%; the optimum range of mean temperature of wettest quarter (bio8) was 7.77°C–9.84°C, peaking at 7.78°C; mean temperature of driest quarter (bio9) had an optimum range of −1.95°C to 6.67°C, peaking at 0.12°C; precipitation seasonality (bio15) had an optimum range of < 32.91 mm; precipitation of coldest quarter (bio19) ranged from 137.36–253.31 mm, peaking at 174.54 mm; and the optimum elevation range was < 159.11 m or > 4513.82 m. The optimum range was < 159.11 m or > 4513.82 m.

### 3.5 Shift in the Centroids of Highly Suitable Habitats Under Three Future Climate Scenarios

**FIGURE 7:**
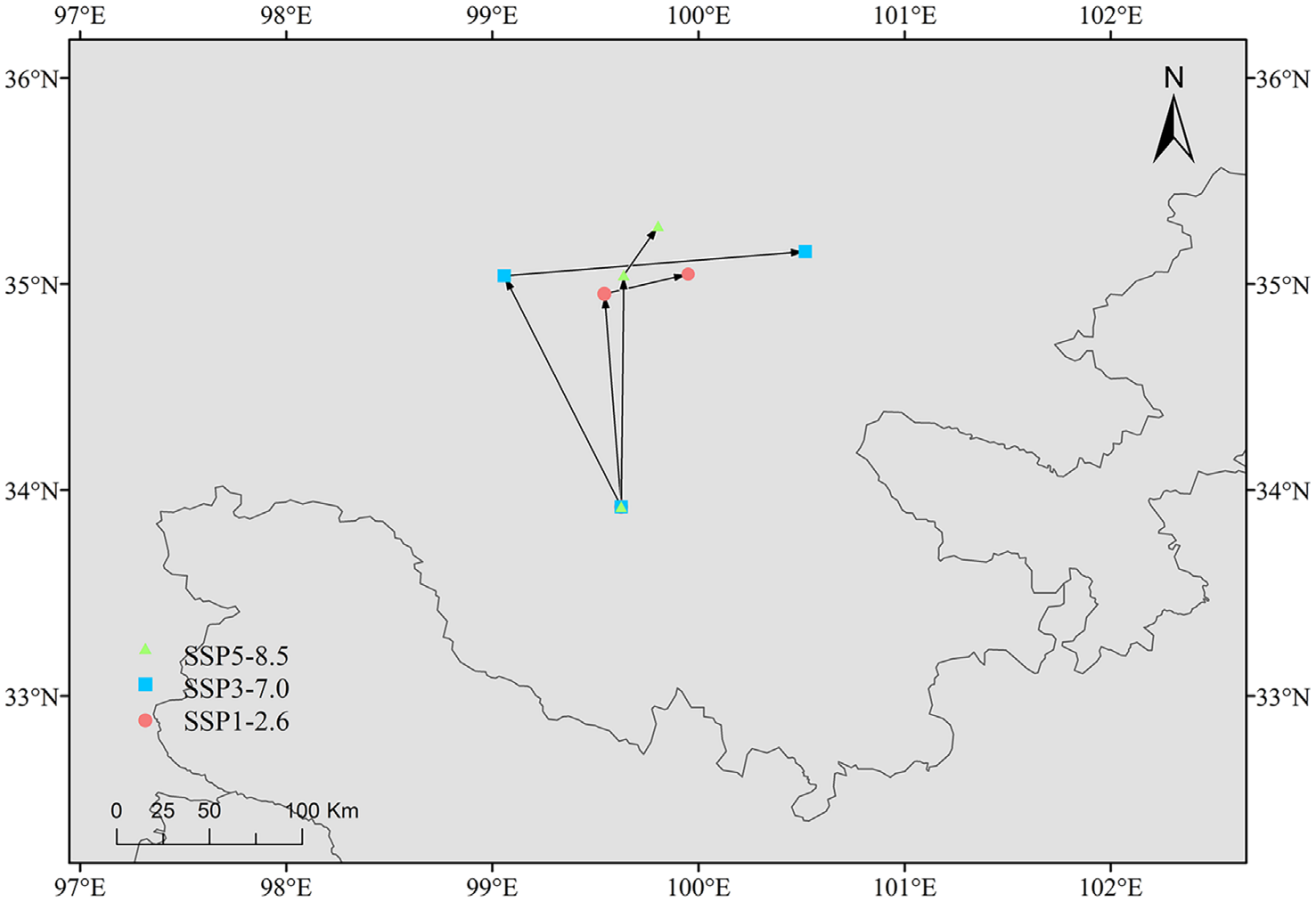
Relocation of highly suitable habitat centers.

Under the current climate scenario, the centroids of highly suitable habitats for *B. cinerea* in China are Gande County, Guoluo Tibetan Autonomous Prefecture, Qinghai Province (99.63° E, 33.92° N). Under the SSP1‐2.6 scenario, the center area of *B. cinerea* 's suitable distribution in China was located in Maqin County, Guoluo Tibetan Autonomous Prefecture, Qinghai Province (99.54° E, 34.95° N) in the 2050s, and in Xinghai County, Hainan Tibetan Autonomous Prefecture, Qinghai Province (99.95° E, 35.05° N) in the 2070s. Under SSP3‐7.0 scenario, the center area of suitable distribution for 2050s *B. cinerea* is located in Maqin County, Guoluo Tibetan Autonomous Prefecture, Qinghai Province (99.06° E, 35.04° N), while 2070s is located in Tongde County, Hainan Tibetan Autonomous Prefecture, Qinghai Province (100.52° E, 35.16° N). Under the SSP5‐8.5 scenario, the center area of suitable distribution of *B. cinerea* in the 2050s was located in Xinghai County, Hainan Tibetan Autonomous Prefecture, Qinghai Province (99.64° E, 35.04° N), and the 2070s was still located in Xinghai County, Hainan Tibetan Autonomous Prefecture, Hainan Province (99.81° E, 35.28° N). It can be seen that the centers of *B. cinerea* 's suitable distributions migrated to higher latitudes in the next six different periods and scenarios, and the centers of *B. cinerea* 's suitable distributions migrated eastward in 2050s and 2070s compared with those in the three scenarios (Figure 7), and the centers of *B. cinerea* 's suitable distributions migrated more in SSP3‐7.0 than in the other two scenarios.

## 4 Discussion

The accuracy of the MaxEnt model is affected by a number of factors, and in this study a number of steps were taken to circumvent factors that might have an impact. The default parameters of the model do not apply to all species, especially when sample sizes are small, and results can vary significantly (Morales San Martin et al. 2017). Therefore, different combinations of FC and RM were evaluated in this paper. The MaxEnt model has the advantage of producing more accurate results even when the sample size of records is small, but the model is more accurate when the sample size exceeds 100 (Xu et al. 2019; Zhuo et al. 2020). Therefore, 138 occurrence records were collected in this paper to ensure the accuracy of the prediction results. In order to avoid the influence of multicollinearity between environmental variables on the simulation results, the environmental variables were screened by VIF and Pearson correlation, and finally, the model results were also evaluated using AUC value. The AUC value was greater than 0.9, which further indicated that our simulation results were reliable.

Fungi are eukaryotic organisms that are estimated to have about 2.2 to 3.8 million species on Earth (Hawksworth and Lücking 2017). Their distribution is influenced by a variety of environmental factors, including temperature, humidity, and light (Baldrian 2016; Peay et al. 2016). Yuan et al. (2015) predicted the potential distribution of three Sanghuang mushrooms (*Phellinus baumii*, *P. igniarius* and *P. vaninii*) using the MaxEnt model, and found that bio13 (precipitation of wettest period), bio16 (precipitation of wettest season) and bio18 (precipitation of warmest season) were the key environmental variables influencing the distribution of Sanghuang mushrooms. The distribution of black mold *Aspergillus niger* is influenced by annual mean temperature (bio1) (Alkhalifah et al. 2022). Wollan et al. (2008) combined the generalized linear model (GLM) with the MaxEnt model and found that temperature was a key factor influencing the distribution of fungi in Norway. It can be seen that temperature and precipitation are important environmental variables affecting the distribution of fungi. Among the environmental variables affecting the distribution of *B. cinerea* in this paper, bio3, bio8, and bio9 were related to temperature, and bio18 and bio19 were related to precipitation. Also, in this paper, elevation was found to be one of the key environmental variables affecting the distribution of *B. cinerea* . The same result was obtained by Ovidiu and Tanase (2017) who found that the optimal habitat distribution of *Ganoderma lucidum* was in mixed forests below an elevation of about 800 m.

Previous studies have shown that *B. cinerea* occurs mainly in temperate and subtropical regions (Williamson et al. 2007). In this paper, in the prediction of the current potential geographic distribution, it was found that the high and medium suitability zones of *B. cinerea* were mainly concentrated in the provinces of Hunan, Hubei, Sichuan, and Shaanxi. According to the climate zoning map of China from the Center for Resource and Environmental Science and Data of the Chinese Academy of Sciences (https://www.resdc.cn/), it can be seen that these provinces are located in the tropical and subtropical monsoon zones, which is in agreement with the results of previous studies. Parts of Qinghai and Xizang are also suitable distribution areas for *B. cinerea* . These two provinces belong to the highland mountainous climate, with an average altitude above 4000 m. The results of the response curves also showed that *B. cinerea* is suitable for distribution at altitudes > 4513.82 m. Future projections indicate that the center of mass of *B. cinerea* 's suitable distribution has not shifted significantly, and the area of the medium‐sized suitable area has shown an increase. According to the IPCC, the global temperature is likely to increase by 1.5°C in the future, which may be the reason for the increase in the area of the mesic zone in the future.

In this work, the effects of multifaceted environmental variables on the distribution of *B. cinerea* were considered in the modeling, including 19 climatic variables and 3 topographic data, but there are still some shortcomings. (Carlile 1965) reported that the light response of the fungus affects spore dispersal. The fungi of the genus *Botrytis* grow in the places where their hosts live (Elad et al. 2006). Therefore, the distribution of hosts can be integrated in subsequent studies. This study still provides insights into understanding the current and future suitable distribution areas of *B. cinerea* and provides a theoretical basis for the prevention of gray mold.

## 5 Conclusion

In this study, the MaxEnt model was used to predict the current potential geographic distribution of *B. cinerea* , and then the suitable distribution areas in the future periods of 2050s and 2070s were predicted by combining the three scenarios of SSP1‐2.6, SSP3‐7.0, and SSP5‐8.5. The results showed that under the current climate scenario in China, the highly suitable area of *B. cinerea* reached 261.15 × 104 km2, which was mainly distributed in the mid‐latitude areas of Central China, East China, Huaihe River Basin, Haihe River Basin, and Xizang Endorheic Region. Under the future climate conditions, the center areas suitable for distribution of *B. cinerea* are all migrating to the high latitude areas, but the migration is not large. Both the current and future center areas suitable for distribution are located in Qinghai Province. In this paper, a total of six environmental variables were screened as key environmental variables affecting the distribution of *B. cinerea* , with climatic factors related to temperature and precipitation, and topographic factors related to altitude. This study contributes to the understanding of the distribution range of *B. cinerea* and helps agricultural managers to take precautionary measures to reduce the risk of disease occurrence. Future studies should focus on integrating real‐time monitoring data with predictive models to enhance the accuracy of disease forecasting systems.

## Author Contributions

**Qianqian Qian:** conceptualization (equal), writing – original draft (equal). **Zhihang Zhuo:** investigation (equal), software (equal), writing – review and editing (equal). **Yaqin Peng:** investigation (equal), software (equal). **Danping Xu:** conceptualization (equal), methodology (equal), writing – review and editing (equal).

## Ethics Statement

The authors have nothing to report.

## Consent

The authors have nothing to report.

## Conflicts of Interest

The authors declare no conflicts of interest.

## Supporting information


**Table S1.** Environmental variables related to the distribution of *Botrytis cinerea*.

## Data Availability

The data supporting the results are available in a public repository at: GBIF.org (01 April 2024) GBIF Occurrence Download https://doi.org/10.15468/dl.nrnjr6.
