# Supplementary material for: Changes in the Distribution of Botrytis cinerea Pers. Fr. In China Under Climate Change
Source: Ecol Evol. 2025 Jul 9;15(7):e71640. doi: 10.1002/ece3.71640 (PMC12240592; doi:10.1002/ece3.71640)
Supplement: Supplementary file 1 — Table S1. Environmental variables related to the distribution of Botrytis cinerea. [file ECE3-15-e71640-s001.docx]

Table S1 Environmental variables related to the distribution of *Botrytis cinerea.*

| Environmental Variable | Description | Unit |
| --- | --- | --- |
| Bio1 | Annual mean temperature | ℃ |
| Bio2 | Mean diurnal range (mean of monthly (max temp - min temp)) | C |
| Bio3 | Isothermality (Bio2/Bio7)(×100) | - |
| Bio4 | Temperature seasonality (standard deviation ×100) | - |
| Bio5 | Maximum temperature of the warmest month | ℃ |
| Bio6 | Minimum temperature of the coldest month | ℃ |
| Bio7 | Temperature annual range (Bio5-Bio6) | ℃ |
| Bio8 | Mean temperature of the wettest quarter | ℃ |
| Bio9 | Mean temperature of the driest quarter | C |
| Bio10 | Mean temperature of the warmest quarter | ℃ |
| Bio11 | Mean temperature of the coldest quarter | ℃ |
| Bio12 | Annual precipitation | mm |
| Bio13 | Precipitation of the wettest month | mm |
| Bio14 | Precipitation of the driest month | mm |
| Bio15 | Precipitation seasonality (coefficient of variation) | - |
| Bio16 | Precipitation of the wettest quarter | mm |
| Bio17 | Precipitation of the driest quarter | mm |
| Bio18 | Precipitation of the warmest quarter | mm |
| Bio19 | Precipitation of the coldest quarter | mm |
| Elev | elevation | m |
| Aspect | - | 。 |
| Slope | - | % |
